# The Q-Matrix Anchored Mixture Rasch Model

**DOI:** 10.3389/fpsyg.2021.564976

**Published:** 2021-03-04

**Authors:** Ming-Chi Tseng, Wen-Chung Wang

**Affiliations:** ^1^National University of Tainan, Tainan, Taiwan; ^2^The Education University of Hong Kong, Tai Po, Hong Kong

**Keywords:** Q-matrix anchored mixture Rasch model, Q-matrix, anchor, mixture Rasch model, Rasch model

## Abstract

Mixture item response theory (IRT) models include a mixture of latent subpopulations such that there are qualitative differences between subgroups but within each subpopulation the measure model based on a continuous latent variable holds. Under this modeling framework, students can be characterized by both their location on a continuous latent variable and by their latent class membership according to Students’ responses. It is important to identify anchor items for constructing a common scale between latent classes beforehand under the mixture IRT framework. Then, all model parameters across latent classes can be estimated on the common scale. In the study, we proposed Q-matrix anchored mixture Rasch model (QAMRM), including a Q-matrix and the traditional mixture Rasch model. The Q-matrix in QAMRM can use class invariant items to place all model parameter estimates from different latent classes on a common scale regardless of the ability distribution. A simulation study was conducted, and it was found that the estimated parameters of the QAMRM recovered fairly well. A real dataset from the Certificate of Proficiency in English was analyzed with the QAMRM, LCDM. It was found the QAMRM outperformed the LCDM in terms of model fit indices.

## Introduction

Measurement invariance is a key assumption that enables score comparison across different groups of respondents (Hambleton et al., 1991). In reality, the assumption may not hold and needs to be checked empirically. In the context of Rasch measurement, different groups of respondents may take different views on items, resulting in measurement non-invariance. Rost (1990) integrated latent class analysis (LCA; Lazarsfeld and Henry, 1968) to the Rasch model (Rasch, 1960) and derived the mixture Rasch model (MRM), which can be viewed as an extension of the Rasch model that allows different groups (latent classes) of respondents to have different item parameters and ability distributions. To the extent that these classes are substantively meaningful, the mixture Rasch model provides a potentially important means to understanding how and why examinees respond in different ways. It is assumed that a Rasch model holds in each class, but each class may have different item and ability parameters. Specifically, the probability of a correct response in the MRM can be given as:

(1) $$P\left(Y_{i\,j}=1\right)=\sum_{g=1}^{G}\pi_{g}\cdot P\left(Y_{i\,j\,g}=1|g,\theta \right)=\sum_{g=1}^{G}\pi_{g}\cdot \frac{\exp\left(\theta_{i\,g}-b_{j\,g}\right)}{1+\exp\left(\theta_{i\,g}-b_{j\,g}\right)},$$

where *g* is an index for the latent class, *g* = 1,…, *G*, *i* = 1,…, *N* examinees, π*_*g*_* is the proportion of examinees for each class, *θ_*ig*_* is the latent ability of examinee *i* in latent class *g*, and *b*_*jg*_ is the difficulty parameter of item *j* for latent class *g*. The MRM can account for qualitative differences between latent classes and quantitative differences within latent classes (Rost, 1990).

An important feature of the MRM or other mixture models (von Davier and Yamamoto, 2007) is that the number of latent classes must be explored from the data, which is an exploratory approach. Usually, the Akaike information criterion (AIC), Bayesian information criterion (BIC), or deviance information criterion (DIC) are applied to determine the number of latent classes but they do not always provide the same answer. Over- or under-extraction of latent classes may occur, making the interpretation problematic (Alexeev et al., 2011). It is desirable to adopt a constrained approach to the identification of latent classes when there are substantive theories or hypotheses.

Recent developments in the Q-matrix (Tatsuoka, 1983) for diagnostic classification models (DCMs) may help with the identification. Domain-specific assessment experts encode the relationships that they believe exist between the diagnostic assessment items and the latent variables that are used to classify respondents into so-called Q-matrices. The attribute is a latent characteristic of respondents in the Q-matrices.

Choi (2010) develop the diagnostic classification mixture Rasch model (DCMixRM) which combines a Mixture Rasch model with log-linear cognitive diagnostic model (LCDM; Henson et al., 2009). In the DCMixRM, this model includes mastery states of attributes as covariates. To be more specific, in the measurement component, observed item responses are jointly regressed on latent trait and attributes through the Rasch model and the LCDM. Next, in the structural model, ability is regressed on class membership, and class membership is regressed on mastery profile to explain latent class as covariates. Besides, Bradshaw and Templin (2014) develop the Scaling Individuals and Classifying Misconceptions (SICM) model which is presented as a combination of a unidimensional IRT model and LCDM where the categorical latent variables represent misconceptions instead of skills. In the SICM, IRT, and LCDM assumed to be orthogonal as in the original bifactor model (Gibbons and Hedeker, 1992). Theoretically, SICM expected that subjects vary in ability even when they possess the same misconception pattern, meaning a significant correlation between ability and misconception pattern was not expected or modeled.

On the other hand, we developed the Q-matrix anchor mixture Rasch model (QAMRM) by incorporating the Q-matrix into the MRM. The QAMRM is constrained because the number of latent classes is specified by users rather than explored from the data. The latent traits in the QAMRM can be compensatory or non-compensatory. The Q-matrix contains a set of elements *q*_*jk*_ indicating whether attribute *k* is required to answer item *j* correctly, and *q*_*jk*_ = 1 if the attribute is required, otherwise it is 0. The total number of attributes and the value of *q*_*jk*_ is assigned by content experts. Similar Q-matrices have been adopted in IRT to specify *a priori* which latent traits (components) have been measured by which items, such as the linear logistic test model (LLTM; Fischer, 1973), the multicomponent latent trait model (Whitely, 1980), the loglinear multidimensional IRT model for polytomously scored items (Kelderman and Rijkes, 1994), the multidimensional random coefficients multinomial logic model (Adams et al., 1997), the multidimensional componential IRT model for polytomous items (Hoskens and De Boeck, 2001), and the multicomponent latent trait model for diagnosis (Embretson and Yang, 2013).

The QAMRM uses the Q-matrix to check whether different classes have different measurement characteristics. The utility of this approach lies in the fact that the numbers of latent classes, immediately observable through the Q-matrix, are defined in advance and can be used to help explain item level performance to discover how members in one class differ from another. It is these Q-matrix differences in response propensities that help explain the potential causes of these differential measurement characteristics.

The approach proposed in this study provides the Q-matrix by means of a design matrix describing the composition of the different classes. We begin below by illustrating how the QAMRM can be viewed as incorporating features from the Q-matrix, and then through the Q-matrix to establish class invariant items and allow all model parameter estimates across latent classes to be on a common scale regardless of the ability distribution.

## The Q-matrix Anchor Mixture Rasch Model

We adapted Figure 1 from Wright and Stone (1979) about the Rasch model to express the response difference between the Rasch model, MRM, and QAMRM.

**FIGURE 1:**
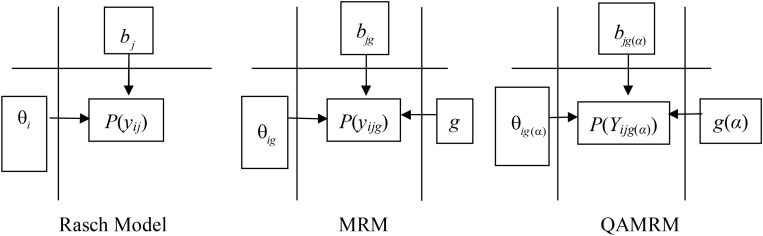
Comparison among the Rasch model, MRM, and QAMRM.

In the Rasch model diagram, person ability *θ_*i*_* and item difficulty *b*_*j*_ jointly determine response *P*(*y*_*ij*_). In the MRM diagram, conditional on latent class *g*, person ability *θ_*ig*_* and item difficulty *b*_*jg*_ jointly determine response *P*(*y*_*ijg*_). In the QAMRM diagram, conditional on class *g*(*α*), person ability *θ_*ig*_*_(_*_α_*_)_ and item difficulty *b*_*jg*__(_*_α_*_)_ jointly determine response *P*(*Y*_*ijg*__(_*_α_*_)_). When there is no Q-matrix, the number of classes is estimated from data, the QAMRM simplifies to the MRM; when there is only single class, the MRM simplifies to the Rasch model.

The QAMRM has multiple classes that follow the Q-matrix design matrix. Like the MRM, it assumes that there may be heterogeneity in response patterns at different classes which should not be ignored (Mislevy and Verhelst, 1990; Rost, 1990), but should consider the Q-matrix to form the number of classes beforehand, rather than forming the number of classes during the parameter estimation. Viewed in this way, the Q-matrix inside the QAMRM captures the association between the items and classes. The probability of getting a correct response in the QAMRM can be given as follows:

(2) $$P\left(Y_{i\,j}=1\right)=\sum_{g\,\left(\alpha \right)=1}^{G}\pi_{g\,\left(\alpha \right)}\cdot P\left(Y_{i\,j\,g\,\left(\alpha \right)}=1|g_{\left(\alpha \right)},\theta_{\left(\alpha \right)}\right)=\sum_{g\,\left(\alpha \right)=1}^{G}\pi_{g\,\left(\alpha \right)}\cdot \frac{\exp\left(\theta_{i\,g\,\left(\alpha \right)}-b_{j\,g\,\left(\alpha \right)}\right)}{1+\exp\left(\theta_{i\,g\,\left(\alpha \right)}-b_{j\,g\,\left(\alpha \right)}\right)},$$

where *Y*_*ijg*__(_*_α_*_)_ is the score of examinee *i* (*i* = 1, …, *N*) on item *j* (*j* = 1, …, *J*) in class *g* conditional on attribute profile *α* (*α* = *α*_1_, …, *α_*K*_*)’, *θ_*ig*_*_(_*_α_*_)_ is the latent ability of examinee *i* within class *g* conditional on attribute *α*, and *b*_*jg*__(_*_α_*_)_ is the difficulty parameter of item *j* for class *g* conditional on attribute *α*. Like the exploratory MRM, there is only one mixing proportion/structural parameter/latent class membership probability in the QAMRM, π*_*g*_*_(_*_α_*_)_, which is the probability of being in class *g* conditional on attribute pattern *α*.

## The Q-matrix Sets Class Invariant Items *A Priori* in QAMRM

Paek and Cho (2015) posit four scenarios to establish a common scale across latent classes in MRM and suggest proposing the use of class-invariant items, which was also suggested by von Davier and Yamamoto (2004). Those invariant items have the same item difficulties across latent classes. Once a set of class invariant items are available, this ensures a common scale across latent classes.

The challenge in MRM is to identify class invariant items, because selecting the best measurement model regarding the numbers of item parameters, latent groups, and dimensions are mainly decided by statistical procedure *post hoc*. Some studies exist in the MRM literature (e.g., Cho et al., 2016), where a statistical procedure was applied as an attempt to locate class invariant items in real data analyses, but how to find class invariant items in the context of MRM until now remains unclear.

Paek and Cho (2015) suggest the use of class invariant items to recover the parameter differences correctly in both item profiles and ability distributions for latent classes when those differences exist simultaneously in MRM; in QAMRM we do not need to find class invariant items by statistical procedure *post hoc* to let all model parameter estimates across latent classes be on a common scale because the Q-matrix has already done that *a prior*. The invariant items in MRM are data driven, but in QAMRM, even though the latent classes follow different ability distributions in terms of their means, we still can easily set invariant items in QAMRM through the Q-matrix *a priori*. Even though there are no ability distributions in the latent class model, through the Q-matrix being set beforehand, the invariance parameters of the DINA model (de la Torre and Lee, 2010) and LCDM model (Bradshaw and Madison, 2016) still hold when the model fit the data.

LCDM is a flexible model that allows the relationships between categorical variables to be modeled using a latent class model, because most cognitive diagnosis models are typically parameterized to define the probability of a correct response, LCDM is re-expressed in terms of the log-odds of a correct response for each item. In addition, von Davier (2005) discusses the General Diagnostic Model (GDM) as a general approach to log-linear models with latent variables, where the latent variables are both continuous and discrete in addition to focusing on ordered responses for items. As a special case, the GDM general definition easily incorporates LCDM with dichotomous latent variables for dichotomous (von Davier, 2014). Besides, Hong et al., 2015 combines DINA model and non-compensatory item response theory to form DINA-NIRT model, which tries to combine continuous and discrete latent variables in cognitive diagnosis. In contrast, the DINA-NIRT is a special case of LCDM because it does not have compensatory attributes. The QAMRM combines continuous and discrete latent variables in the same framework, which includes compensatory (disjunctive) and non-compensatory (conjunctive) models.

In compensatory QAMRM, a low value on one latent variable can be compensated for by a high value on another latent variable, so it is not necessary to master all attributes that are required by an item to produce a correct response within a class. On the contrary, in non-compensatory QAMRM, a low value on one latent variable cannot be compensated for by a high value on another latent variable, so it is necessary to master all attributes that are required by an item to produce a correct response within a class. In the QAMRM, the relationship among latent variables, either compensatory or non-compensatory, is assumed to be identical across classes.

Substantive theories can help decide the numbers of attributes and classes prior to parameter estimation, as done in the Q-matrix. As an example, let there be 14 items measuring three binary attributes in the QAMRM. In total, there will be eight (2^3^) attribute profiles, which are called classes *g*_1_-*g*_8_ as shown in column 1 in Table 1.

**TABLE 1:** Class, Q-matrix and item difficulty for 14-item 3 attributes QAMRM.

**Class**				**Q-matrix**				**Non-compensatory**									**Compensatory**								
**1**	**2**	**3**	**4**	**5**	**6**	**7**	**8**	**9**	**10**	**11**	**12**	**13**	**14**	**15**	**16**	**17**	**18**	**19**	**20**	**21**	**22**	**23**	**24**	**25**	**26**
			
	***α*_1_**	***α*_2_**	***α*_3_**	**Item**	***α*_1_**	***α*_2_**	***α*_3_**	**Item**	***g*_1_**	***g*_2_**	***g*_3_**	***g*_4_**	***g*_5_**	***g*_6_**	***g*_7_**	***g*_8_**	**Item**	***g*_1_**	***g*_2_**	***g*_3_**	***g*_4_**	***g*_5_**	***g*_6_**	***g*_7_**	***g*_8_**
*g*_1_	0	0	0	1	1	0	0	1	2^*a*^	2	2	2	1	1	1	1	1	2	2	2	2	1	1	1	1
*g*_2_	0	0	1	2	0	1	0	2	2	2	1	1	2	2	1	1	2	2	2	1	1	2	2	1	1
*g*_3_	0	1	0	3	0	0	1	3	2	1	2	1	2	1	2	1	3	2	1	2	1	2	1	2	1
*g*_4_	0	1	1	4	1	1	0	4	2	2	2	2	2	2	1	1	4	3	3	2	2	2	2	1	1
*g*_5_	1	0	0	5	1	0	1	5	2	2	2	2	2	1	2	1	5	3	2	3	2	2	1	2	1
*g*_6_	1	0	1	6	0	1	1	6	2	2	2	1	2	2	2	1	6	3	2	2	1	3	2	2	1
*g*_7_	1	1	0	7	1	1	1	7	2	2	2	2	2	2	2	1	7	4	3	3	2	3	2	2	1
*g*_8_	1	1	1	8	1	0	0	8	2	2	2	2	1	1	1	1	8	2	2	2	2	1	1	1	1
				9	0	1	0	9	2	2	1	1	2	2	1	1	9	2	2	1	1	2	2	1	1
				10	0	0	1	10	2	1	2	1	2	1	2	1	10	2	1	2	1	2	1	2	1
				11	1	1	0	11	2	2	2	2	2	2	1	1	11	3	3	2	2	2	2	1	1
				12	1	0	1	12	2	2	2	2	2	1	2	1	12	3	2	3	2	2	1	2	1
				13	0	1	1	13	2	2	2	1	2	2	2	1	13	3	2	2	1	3	2	2	1
				14	1	1	1	14	2	2	2	2	2	2	2	1	14	4	3	3	2	3	2	2	1

*${}^{a}$The 2 represents the label we are using for the threshold in Item 1. Therefore, the whole line to label the first threshold of Item 1 is 2. More importantly, each parameter with a label of 2 in Item 1 will be equated, thus providing the same value for the estimated threshold. The label 2 between each item is different; the 2 is the label, not the number.*

Columns 2–4 give the attribute profiles for *g*_1_-*g*_8_. Persons in *g*_1_ (0,0,0) have not mastered any of the three attributes; persons in *g*_2_ (0,0,1) have mastered *α*_3_ but have not mastered *α*_1_ and *α*_2_; and so on for the other classes. Note that we follow substantive theories to set *g* classes conditional on Q-matrix (*2*^*K*^) when saturated by QAMRM *a priori*, if the class has almost no examinees in practice (de la Torre and Lee, 2010; Templin and Bradshaw, 2014), and we can thus cancel out the class.

Columns 6–8 show the Q-matrix for the 14 items. For example, item 1 measures *α*_1_, item 4 measures *α*_1_ and *α*_2_, item 14 measures *α*_1_, *α*_2_, and *α*_3_.

Columns 10–17 list hypothetical difficulties of the 14 items for the eight classes when the attributes are non-compensatory. For example, the difficulty of item 1 is 2 for *g*_1_-*g*_4_ but 1 for *g*_5_-*g*_8_. Because persons in g_1_-*g*_4_ have not mastered the attribute that item 1 measures (*α*_1_), the item difficulty for them would be equally high. On the contrary, persons in *g*_5_-*g*_8_ have mastered *α*_1_, so the item difficulty for them would be equally low. That is, although there are eight classes, item 1 has only two difficulties, one for *g*_1_-*g*_4_ and the other for *g*_5_-*g*_8_. Likewise, item 4 have difficulty of 2 for *g*_1_-*g*_6_ but 1 for *g*_7_-*g*_8_. Persons in *g*_1_-*g*_6_ have not mastered all attributes that are measured by item 4 (*α*_1_ and *α*_2_) so the item difficulty for them would be equally high; on the contrary, persons in *g*_7_-*g*_8_ have mastered both *α*_1_ and *α*_2_ so the item difficulty for them would be equally low. The other items can be interpreted similarity.

Columns 19–26 list hypothetical difficulties of the 14 items for the eight classes when the attributes are compensatory. Item 4 have difficulty of 3 for *g*_1_ and *g*_2_, and 2 for *g*_3_-*g*_6_, and 1 for *g*_7_ and *g*_8_. Persons in *g*_1_ and *g*_2_ have not mastered any of the attributes that item 4 measures (*α*_1_ and *α*_2_) so the item difficulty for them would be equally high; persons in *g*_3_-*g*_6_ have mastered one of *α*_1_ and *α*_2_ so the item difficulty for them would be equally median; persons in *g*_7_ and *g*_8_ have mastered both *α*_1_ and *α*_2_ so the item difficulty for them would be equally low. In other words, although there are eight class, item 4 has three difficulties, one for *g*_1_ and *g*_2_, one for *g*_3_-*g*_6_, and the other for *g*_7_ and *g*_8_, and the three difficulties are expected to be ordered. The other items can be interpreted similarity.

The 14 item parameters were specified according to the Q-matrix. The Q-matrix is like a bridge to connect different latent classes together and sets item parameter constraints across different latent classes *a priori*, like anchor items in different latent classes, hence the Q-matrix in QAMRM can be considered as the “anchor attribute.”

For illustrative purposes, the item difficulties in the Table 1 are set as integers, when in reality, they can be real numbers. However, the ordinal nature of 4 > 3 > 2 > 1 is expected. If the Q-matrix was not adopted, there are 112 item parameters (assuming there were eight latent classes and 14 item parameters in each latent class), while according to the Q-matrix, in the non-compensatory model, each of the 14 items has two difficult parameters for the eight classes, so the total number of difficulty parameters is 28, in the compensatory model, six, six, and two items have two, three, and four difficult parameters for the eight classes, respectively, so the total number of difficulty parameters is 38.

## Simulation Study

The primary goal of this section is to demonstrate that when the QAMRM fits the item responses, through the Q-matrix setting invariant items, all model parameter estimates across latent classes are on a common scale and will be invariant regardless of the nature of the latent ability distributions across latent classes.

In the mixture Rasch model for binary data as described by Rost (1990) who used the model constraint _∑δ_ig = 0_ in MRM, where _δ_ig_ the item difficulty of the *i*th item is in the *g*th latent class and the summation is over items at a given *g*, indicating the summation of group specific item difficulty parameters over items is 0 within a latent group, and compared item profiles to characterize latent groups. The constraint can be used for scale comparability only when there is no mean difference in a continuous latent variable. Because we do not know the “true” mean difference in real data sets, the constraint cannot be sufficient for all empirical data sets (Cho et al., 2016). An alternative to identifying a common scale is by making the mean of the item parameters on latent ability zero (Wu et al., 1998), and when calculating every item difficulty parameter in latent classes conditional on Q-matrix, the QAMRM uses this setting.

Several aspects of the simulated data were held constant: the number of attributes was fixed to *K* = 3, test length to *J* = 14; the item parameters were generated as follows. For non-compensatory QAMRM (in Table 1 columns 10–17), each item had two levels of difficulty and the item parameter was set at either −2 or 2. For compensatory QAMRM (in Table 1 columns 19–26), an item could have 2–4 levels of difficulty. When there were two levels (items 1–3, 8–10), the item parameter was set at −2 or 2; When there were three levels (items 4–6, 11–13), the item parameter was set at −2, 1, or 2. When there were four levels (items 7, 14), the item parameter was set at −2, −1, 1, or 2. The eight classes were uniformly distributed, meaning that the mixing proportion for each class was 12.5%. There were three sample sizes (1,000, 2,000, and 4,000) for the latent ability distributions that involve the QAMRM model, with ability distributions with means _μ_θ = 0.0_ and a common standard deviation _σ_θ = 1.0_ been used. Note that the mixture Rasch model is employed here for discussion, where the slope parameters of items in the item response function is unity. Therefore, the difference in _σ_g^2_ does not pose a problem in establishing a common scale between latent classes (Paek and Cho, 2015).

A total of 1,000 replications were generated under each condition. The item parameters were estimated via an EM implementation of the marginal maximum likelihood estimation (MMLE/EM) that was implemented using the computer program Mplus (Muthén and Muthén, 2021). In the EM estimation, maximum likelihood optimization was done in two stages. In the initial stage, 20 random sets of initial values were generated. An optimization was carried out for 10 iterations using each of the 20 random sets of initial values. The final values from the four optimizations with the highest log-likelihoods were used as the starting values in the final stage optimizations (Muthén and Muthén, 2021). The problems with the local maximum in the QAMRM did not occur because the number of classes was specified by the user rather than explored from the data as in finite mixture models.

The parameters in QAMRM included the mixing proportion, latent class membership conditional on the attribute profile specific item parameters, and the population parameters of latent class conditional on the attribute profile specific continuous latent variable. In the QAMRM, the Q-matrix was adopted to constrain the item parameters to be invariant across latent classes, which could reduce a large number of item parameters and improve parameter estimation (see Table 1). Take non-compensatory QAMRM in the simulation as an example, the total parameters to be estimated were 36, including seven mixing proportion parameters (the mixing proportion parameters should add up to 1, so only seven parameters could be estimated when there were three latent attributes and eight latent profiles), one variance parameter, and 28 item parameters for the 14 items which were specified according to the Q-matrix. If the Q-matrix was not adopted, the model became the MRM, which would estimate 120 parameters, including 112 item parameters (assuming there were eight latent classes and 14 item parameters in each latent class), seven mixing proportion parameters, and one variance parameter. Such a large number of parameters would require a large sample size, which would be a practical constraint.

To evaluate the parameter recovery, we computed the bias, the 95% coverage rate for the item parameter estimates in Table 2. Due to enhance readability, we do not report bias and coverage rate for individual parameters; rather, we show the mean bias and mean coverage rate across all parameters in Table 2.

**TABLE 2:** Bias and 95% coverage rate for the item parameters in the simulation study.

**Sample size**	**Non-compensatory**		**Compensatory**	
	**Bias**	**Coverage**	**Bias**	**Coverage**
1,000	0.01	0.95	0.00	0.96
2,000	0.00	0.95	0.00	0.95
4,000	0.00	0.95	0.00	0.95

*The detail simulation results about bias and coverage rate for individual parameters can be found through the link: https://www.dropbox.com/sh/b3sb3ju4swk1pmi/AABpbXMRLXFji52PfHmNbi0Za?dl=0.*

The EM estimation method yielded very small bias. Besides, the mean coverage was very close to 95%. The results of the simulated data analysis indicate that the invariance property of the QAMRM model is absolute in that the parameter estimates were obtained using different calibration samples. By means of the Q-matrix setting invariant items *a priori* in QAMRM, all model parameter estimates across latent classes to be on a common scale, which does not require any transformation for them to be comparable.

## Real Data Analysis

We used the Certificate of Proficiency in English (ECPE) data, which is available in the R package CDM (Robitzsch et al., 2011–2014), to demonstrate the advantages of the QAMRM over the LCDM. The ECPE data consist of responses from 2,922 test-takers to 28 items, with each item measuring one or two out of three skills. The data has been analyzed with the LCDM by Templin and Hoffman (2013) and Templin and Bradshaw (2014) and with the GDM by von Davier (2014). As shown previously, when analyze the ECPE data, the LCDM and GDM are mathematically equivalent (von Davier, 2014), hence we fit the QAMRM to the data using Mplus and compared the results with those under the LCDM (Templin and Hoffman, 2013; Templin and Bradshaw, 2014).

The Q-matrix used in ECPE example was the result of psychometric analyses on the ECPE by Buck and Tatsuoka (1998). The analyses showed that items of the test were likely to measure three distinct skills. The left side of Table 3 shows the skill profile and the right side of Table 3 shows Q-matrix that maps each item to the three skills. As shown, eight items measure only one skill, seven items measure two skills, and zero items measure three skills. The morphosyntactic (*α*_1_), cohesive (*α*_2_), and lexical (*α*_3_) skills were each measured by 13, 6, and 18 items, respectively.

**TABLE 3:** ECPE Q-Matrix and the skill profile.

	**Skill profile (*α*_1_, *α*_2_, *α*_3_)**				**Q-matrix**		
**Class**	***α*_1_**	***α*_2_**	***α*_3_**	**Item**	***α*_1_**	***α*_2_**	***α*_3_**
*g*_1_	0	0	0	1	1	1	0
*g*_2_	0	0	1	2	0	1	0
*g*_3_	0	1	0	3	1	0	1
*g*_4_	0	1	1	4	0	0	1
*g*_5_	1	0	0	5	0	0	1
*g*_6_	1	0	1	6	0	0	1
*g*_7_	1	1	0	7	1	0	1
*g*_8_	1	1	1	8	0	1	0
				9	0	0	1
				10	1	0	0
				11	1	0	1
				12	1	0	1
				13	1	0	0
				14	1	0	0
				15	0	0	1
				16	1	0	1
				17	0	1	1
				18	0	0	1
				19	0	0	1
				20	1	0	1
				21	1	0	1
				22	0	0	1
				23	0	1	0
				24	0	1	0
				25	1	0	0
				26	0	0	1
				27	1	0	0
				28	0	0	1

*α_1_, Morphosyntactic rules; α_2_, Cohesive rules; α_3_, Lexical rules.*

Therefore, we only report the skill distributions with latent class pattern and model fit results with the values published by Templin and Hoffman (2013) and Templin and Bradshaw (2014) which agree with those obtained from the CDM R-package (Robitzsch et al., 2011–2014).

We use AIC, BIC, and sample-size adjusted BIC (ABIC) to select the best model (Templin and Bradshaw, 2014), the information criteria selected the best model by small value. Table 4 presents AIC, BIC, and ABIC for the QAMRM, LCDM. It appears that the QAMRM had lower AIC (85131.55–85641.43), BIC (85568.09–86125.81), and ABIC (85336.14–85868.44). Table 5 shows the distributions of the eight skill profiles (classes) obtained from the QAMRM, LCDM. The distributions were very similar across models and only four skill profiles were substantial: (0,0,0), (1,0,0), (1,1,0), and (1,1,1).

**TABLE 4:** Comparisons of model-data fit among the QAMRM, LCDM.

**Model**	**AIC**	**BIC**	**ABIC**
LCDM^*a*^	85641.43	86125.81	85868.44
QAMRM	85131.55	85568.09	85336.14
Hierarchical LCDM^*b*^	85638.63	86045.08	85829.21
Hierarchical QAMRM	85125.80	85538.42	85319.18

*^*a*^LCDM = log-linear cognitive diagnostic model; More details on these models can be found in Templin and Hoffman (2013).*

*^*b*^Hierarchical LCDM; More details on these models can be found in Templin and Bradshaw (2014).*

**TABLE 5:** Skill profile distributions obtained for the QAMRM, LCMD.

**Skill profile**	**QAMRM**	**LCDM^*a*^**	**Skill profile**	**Hierarchical LCDM^*b*^**	**Hierarchical QAMRM**
(0,0,0)	0.44	0.30	(0,0,0)	0.34	0.44
(0,0,1)	0.11	0.13	(0,0,1)	0.11	0.08
(0,1,0)	0.00	0.01	(0,1,1)	0.18	0.13
(0,1,1)	0.10	0.18	(1,1,1)	0.38	0.34
(1,0,0)	0.00	0.01			
(1,0,1)	0.01	0.02			
(1,1,0)	0.00	0.01			
(1,1,1)	0.33	0.35			

*^*a*^Templin and Hoffman (2013).*

*^*b*^Templin and Bradshaw (2014).*

Templin and Hoffman (2013) analyzed a sample of 2,922 examinees who took the ECPE with the non-hierarchical LCDM. But in the ECPE example, the data and results suggest a linear attribute hierarchy: Examinees must master Attribute 3 (lexical rules) before mastering Attribute 2 (cohesive rules) before mastering Attribute 1 (morphosyntactic rules). Gierl et al. (2007) call this structure a linear hierarchy, where mastery of each attribute follows a linear progression. Therefore, Templin and Bradshaw (2014) introduce the hierarchical LCDM where attribute hierarchies are present, the model fit of hierarchical LCDM shown at the bottom of Table 4, the hierarchical LCDM is used to test for the presence of a suspected attribute hierarchy in ECPE, through model fit which confirming the data is more adequately represented by hierarchical attribute structure when compared to a crossed, or non-hierarchical structure.

We reanalyzed the data with hierarchical QAMRM, and compare the model fit with hierarchical LCDM. Right column of Table 5 shows the distributions of the four hierarchical skill profiles (classes) obtained from the hierarchical QAMRM, hierarchical LCDM. It also appears that the hierarchical QAMRM had lower AIC (85125.80–85638.63), BIC (85538.42–86045.08), and ABIC (85319.18–85829.21).

Table 6 presents Q-matrix in ECPE and hierarchical QAMRM item difficulty parameter estimates. In items 4–10,13–15, 18–19, 22–28 only one attribute is measured, with all of these items inside hierarchical QAMRM non-compensatory, having only two different kinds of item difficulty; take item 4 for example; if the examiner masters attribute *α*_3_, he will have a high probability to answer item 4 correctly, with the item difficulty being −2.54, while, on the other hand, if the examiner does not master attribute *α*_3_, he will have a low probability to answer the item correct, with the item difficulty becoming −0.31, and the other items can be interpreted similarity.

**TABLE 6:** ECPE Q-matrix and hierarchical QAMRM item difficulty parameter estimates.

**Item**		**Skill**		**Skill profile (α_1_, α_2_, α_3_)**			
	***α*_1_**	***α*_2_**	***α*_3_**	**(0,0,0)**	**(0,0,1)**	**(0,1,1)**	**(1,1,1)**
1	1	1	0	−1.98	−1.98	−2.04	−3.99
2	0	1	0	−2.30	−2.30	−3.42	−3.42
3	1	0	1	−0.01	−0.01	−0.01	−2.08
4	0	0	1	−0.31	−2.54	−2.54	−2.54
5	0	0	1	−2.27	−5.14	−5.14	−5.14
6	0	0	1	−1.95	−4.18	−4.18	−4.18
7	1	0	1	−0.43	−1.58	−1.58	−4.98
8	0	1	0	−3.09	−3.09	−4.93	−4.93
9	0	0	1	−0.68	−2.14	−2.14	−2.14
10	1	0	0	−0.42	−0.42	−0.42	−3.73
11	1	0	1	−0.42	−1.86	−1.86	−4.10
12	1	0	1	2.68	0.30	0.30	−1.98
13	1	0	0	−1.45	−1.45	−1.45	−3.73
14	1	0	0	−0.68	−0.68	−0.68	−2.26
15	0	0	1	−2.20	−5.01	−5.01	−5.01
16	1	0	1	−0.37	−1.62	−1.62	−3.77
17	0	1	1	−2.94	−2.94	−4.54	−4.54
18	0	0	1	−2.11	−3.70	−3.70	−3.70
19	0	0	1	−0.14	−2.83	−2.83	−2.83
20	1	0	1	1.88	0.49	0.49	−2.16
21	1	0	1	−0.83	−2.46	−2.46	−3.69
22	0	0	1	1.06	−2.56	−2.56	−2.56
23	0	1	0	−1.62	−1.62	−4.31	−4.31
24	0	1	0	0.46	0.46	−1.12	−1.12
25	1	0	0	−0.50	−0.50	−0.50	−1.79
26	0	0	1	−0.86	−1.98	−1.98	−1.98
27	1	0	0	1.09	1.09	1.09	−1.34
28	0	0	1	−1.35	−3.97	−3.97	−3.97

*The syntax and detail analysis results can be found in: https://www.dropbox.com/sh/b3sb3ju4swk1pmi/AABpbXMRLXFji52PfHmNbi0Za?dl=0.*

*For items 1, 3, 11–12, 16–17, and 20–21, by means of the Q-matrix we can see all of these items measuring two attributes, with those inside QAMRM compensatory. Take item 1 for example; item 1 measures attributes α_1_ and α_2_; if the examiner masters attribute α_1_ and α_2_, he will have a high probability to answer the item 1 correctly, with the item difficulty in item 1 being*−*3.99, while if the examiner does not master attributes α_1_ and α_2_ at the same time, and just masters attribute α_2_ compensatorily, he will have a moderate probability to answer the item correctly, with the item difficulty becoming*−*2.04. However, if the examiner does not master attributes α_1_ and α_2_, he will have a low probability of answering the item correctly, with the item difficulty becoming*−*1.98. The other items can be interpreted similarly.*

*If the examiner doesn’t master any skill, he will fall into latent class (α_1_, α_2_, α_3_) = (0, 0, 0) and will have the lowest probability of correctly answering all the items. If the examiner just masters the α_3_ skill, but does not master the α_1_ and α_2_ skills, the examiner will fall into (α_1_, α_2_, α_3_) = (0, 0, 1), and the examine will have a higher probability of correctly answering items 4–6, 9, 15, 18–19, 22, 26, 28 than the examiner who hasn’t mastered the α_3_ skill. The details can be seen in Table 6. The other items can be interpreted similarly.*

If we only use the LCDM or Hierarchical LCDM for analysis, a second calculation is still needed to find the probability of a correct response for each item, which may not easy for practitioners.

## Conclusion and Discussion

The QAMRM was used to describe for modeling the Q-matrix at the mixture Rasch model. The model developed in this study used features of a Rasch model, a restricted latent class model, and a Q-matrix. The Q-matrix of the model provides an opportunity to determine the number of latent class in advance through substantive theory and not through model fitness or parameter estimation *post hoc*. Information in the Q-matrix can be used to reveal possible differences that might be due to differences among latent classes.

A simulation study through the EM algorithm estimation was presented to investigate the performance of the model. Generated parameters were well recovered for the conditions considered. The QAMRM makes it possible to describe the differential item performance of target attributes using descriptions of Q-matrix characteristics associated with the items compared with characteristics associated with other items not in the same latent classes. This description can then be used to provide the Q-matrix with a framework within which to compare the results in their latent classes and in the other latent classes. Examiners in each of the latent class can be characterized by differences in attribute, as well as by differences in response strategies, particularly at the end of the test.

The real data comparison performed between the QAMRM, LCDM, by means of the ECPE data, shows that when a Rasch model is included inside the diagnostic classification models, QAMRM achieves a more desirable result and has better fit indices than the LCDM variants.

If we want to provide a single, continuous estimate of overall ability and classify the subjects at the same time, we should consider the QAMRM rather than mixture Rasch model or DCM. In QAMRM, the Q-matrix sets class invariant items a priori, if the Q-matrix design is not correct, then the analysis in the QAMRM will be wrong. Kopf et al. (2015) who have discussed anchoring strategies in details which can help to correct the Q-matrix design. Future research can focus on the misspecify Q-matrix design with the model unfit of the QAMRM.

On the other hand, future research can focus on the estimation limitations of the QAMRM. Specifically, as the number of attributes included in the Q-matrix increases and as its complexity increases, the number of parameters estimated by this model will also increase. In these cases, expectations of its performance in estimated attribute mastery and item parameters must be explored. In addition, model comparisons using common indices such as the AIC, BIC, and ABIC must continue to be explored, which could result to clear guidelines for model identification. Finally, possible expansions of this model such as the addition of a continuous ability measure to imply an incomplete Q-matrix (much like what is used in the testlet IRT) will be explored.

## Data Availability Statement

Publicly available datasets were analyzed in this study. This data can be found here: http://cran.r-project.org/web/packages/CDM/index.html.

## Author Contributions

M-CT: writing and analyzing, 80%. W-CW: correction and feedback, 20%. Both authors contributed to the article and approved the submitted version.

## Conflict of Interest

The authors declare that the research was conducted in the absence of any commercial or financial relationships that could be construed as a potential conflict of interest.
