# Supplementary material for: The Q-Matrix Anchored Mixture Rasch Model
Source: Front Psychol. 2021 Mar 4;12:564976. doi: 10.3389/fpsyg.2021.564976 (PMC7969527; doi:10.3389/fpsyg.2021.564976)
Supplement: Supplementary file 1 [file Data_Sheet_1.PDF]

Appendix A. EM Estimation with the Noncompensatory QAMRM (N = 1000)

```
title:
montecarlo:
        NAMES = x1-x14;
        generate = x1-x14(1);
        categorical = x1-x14;
        genclasses = c(8);
        classes = c(8);
        nobs = 1000;
        seed = 3454367;
        nrep = 1000;


ANALYSIS:
        TYPE = MIXTURE;
       ALGORITHM=INTEGRATION;

model population:
        %overall%
       f BY x1-x14*1(1);
       [f@0];
       f@1;
        [c#1*0];
        [c#2*0];
        [c#3*0];
        [c#4*0];
        [c#5*0];
        [c#6*0];
        [c#7*0];

%c#1%
[x1$1*2](t1_1);
[x2$1*2](t2_1);
[x3$1*2](t3_1);
[x4$1*2](t4_1);
[x5$1*2](t5_1);
[x6$1*2](t6_1);
[x7$1*2](t7_1);
[x8$1*2](t8_1);
[x9$1*2](t9_1);
[x10$1*2](t10_1);
[x11$1*2](t11_1);
[x12$1*2](t12_1);
[x13$1*2](t13_1);
[x14$1*2](t14_1);
%c#2%
[x1$1*2](t1_1);
[x2$1*2](t2_1);
[x3$1*-2](t3_2);
[x4$1*2](t4_1);
[x5$1*2](t5_1);
[x6$1*2](t6_1);
[x7$1*2](t7_1);
[x8$1*2](t8_1);
[x9$1*2](t9_1);
[x10$1*-2](t10_2);
[x11$1*2](t11_1);
```

```
[x12$1*2](t12_1);
[x13$1*2](t13_1);
[x14$1*2](t14_1);
%c#3%
[x1$1*2](t1_1);
[x2$1*-2](t2_2);
[x3$1*2](t3_1);
[x4$1*2](t4_1);
[x5$1*2](t5_1);
[x6$1*2](t6_1);
[x7$1*2](t7_1);
[x8$1*2](t8_1);
[x9$1*-2](t9_2);
[x10$1*2](t10_1);
[x11$1*2](t11_1);
[x12$1*2](t12_1);
[x13$1*2](t13_1);
[x14$1*2](t14_1);
%c#4%
[x1$1*2](t1_1);
[x2$1*-2](t2_2);
[x3$1*-2](t3_2);
[x4$1*2](t4_1);
[x5$1*2](t5_1);
[x6$1*-2](t6_2);
[x7$1*2](t7_1);
[x8$1*2](t8_1);
[x9$1*-2](t9_2);
[x10$1*-2](t10_2);
[x11$1*2](t11_1);
[x12$1*2](t12_1);
[x13$1*-2](t13_2);
[x14$1*2](t14_1);
%c#5%
[x1$1*-2](t1_2);
[x2$1*2](t2_1);
[x3$1*2](t3_1);
[x4$1*2](t4_1);
[x5$1*2](t5_1);
[x6$1*2](t6_1);
[x7$1*2](t7_1);
[x8$1*-2](t8_2);
[x9$1*2](t9_1);
[x10$1*2](t10_1);
[x11$1*2](t11_1);
[x12$1*2](t12_1);
[x13$1*2](t13_1);
[x14$1*2](t14_1);
%c#6%
[x1$1*-2](t1_2);
[x2$1*2](t2_1);
[x3$1*-2](t3_2);
[x4$1*2](t4_1);
[x5$1*-2](t5_2);
[x6$1*2](t6_1);
[x7$1*2](t7_1);
[x8$1*-2](t8_2);
[x9$1*2](t9_1);
```

```
[x10$1*-2](t10_2);
[x11$1*2](t11_1);
[x12$1*-2](t12_2);
[x13$1*2](t13_1);
[x14$1*2](t14_1);
%c#7%
[x1$1*-2](t1_2);
[x2$1*-2](t2_2);
[x3$1*2](t3_1);
[x4$1*-2](t4_2);
[x5$1*2](t5_1);
[x6$1*2](t6_1);
[x7$1*2](t7_1);
[x8$1*-2](t8_2);
[x9$1*-2](t9_2);
[x10$1*2](t10_1);
[x11$1*-2](t11_2);
[x12$1*2](t12_1);
[x13$1*2](t13_1);
[x14$1*2](t14_1);
%c#8%
[x1$1*-2](t1_2);
[x2$1*-2](t2_2);
[x3$1*-2](t3_2);
[x4$1*-2](t4_2);
[x5$1*-2](t5_2);
[x6$1*-2](t6_2);
[x7$1*-2](t7_2);
[x8$1*-2](t8_2);
[x9$1*-2](t9_2);
[x10$1*-2](t10_2);
[x11$1*-2](t11_2);
[x12$1*-2](t12_2);
[x13$1*-2](t13_2);
[x14$1*-2](t14_2);

model:
      %overall%
     f BY x1-x14*1(1);
     [f@0];
     f@1;
      [c#1*0];
      [c#2*0];
      [c#3*0];
      [c#4*0];
      [c#5*0];
      [c#6*0];
      [c#7*0];

%c#1%
[x1$1*2](t1_1);
[x2$1*2](t2_1);
[x3$1*2](t3_1);
[x4$1*2](t4_1);
[x5$1*2](t5_1);
[x6$1*2](t6_1);
[x7$1*2](t7_1);
[x8$1*2](t8_1);
```

```
[x9$1*2](t9_1);
[x10$1*2](t10_1);
[x11$1*2](t11_1);
[x12$1*2](t12_1);
[x13$1*2](t13_1);
[x14$1*2](t14_1);
%c#2%
[x1$1*2](t1_1);
[x2$1*2](t2_1);
[x3$1*-2](t3_2);
[x4$1*2](t4_1);
[x5$1*2](t5_1);
[x6$1*2](t6_1);
[x7$1*2](t7_1);
[x8$1*2](t8_1);
[x9$1*2](t9_1);
[x10$1*-2](t10_2);
[x11$1*2](t11_1);
[x12$1*2](t12_1);
[x13$1*2](t13_1);
[x14$1*2](t14_1);
%c#3%
[x1$1*2](t1_1);
[x2$1*-2](t2_2);
[x3$1*2](t3_1);
[x4$1*2](t4_1);
[x5$1*2](t5_1);
[x6$1*2](t6_1);
[x7$1*2](t7_1);
[x8$1*2](t8_1);
[x9$1*-2](t9_2);
[x10$1*2](t10_1);
[x11$1*2](t11_1);
[x12$1*2](t12_1);
[x13$1*2](t13_1);
[x14$1*2](t14_1);
%c#4%
[x1$1*2](t1_1);
[x2$1*-2](t2_2);
[x3$1*-2](t3_2);
[x4$1*2](t4_1);
[x5$1*2](t5_1);
[x6$1*-2](t6_2);
[x7$1*2](t7_1);
[x8$1*2](t8_1);
[x9$1*-2](t9_2);
[x10$1*-2](t10_2);
[x11$1*2](t11_1);
[x12$1*2](t12_1);
[x13$1*-2](t13_2);
[x14$1*2](t14_1);
%c#5%
[x1$1*-2](t1_2);
[x2$1*2](t2_1);
[x3$1*2](t3_1);
[x4$1*2](t4_1);
[x5$1*2](t5_1);
[x6$1*2](t6_1);
```

```
[x7$1*2](t7_1);
[x8$1*-2](t8_2);
[x9$1*2](t9_1);
[x10$1*2](t10_1);
[x11$1*2](t11_1);
[x12$1*2](t12_1);
[x13$1*2](t13_1);
[x14$1*2](t14_1);
%c#6%
[x1$1*-2](t1_2);
[x2$1*2](t2_1);
[x3$1*-2](t3_2);
[x4$1*2](t4_1);
[x5$1*-2](t5_2);
[x6$1*2](t6_1);
[x7$1*2](t7_1);
[x8$1*-2](t8_2);
[x9$1*2](t9_1);
[x10$1*-2](t10_2);
[x11$1*2](t11_1);
[x12$1*-2](t12_2);
[x13$1*2](t13_1);
[x14$1*2](t14_1);
%c#7%
[x1$1*-2](t1_2);
[x2$1*-2](t2_2);
[x3$1*2](t3_1);
[x4$1*-2](t4_2);
[x5$1*2](t5_1);
[x6$1*2](t6_1);
[x7$1*2](t7_1);
[x8$1*-2](t8_2);
[x9$1*-2](t9_2);
[x10$1*2](t10_1);
[x11$1*-2](t11_2);
[x12$1*2](t12_1);
[x13$1*2](t13_1);
[x14$1*2](t14_1);
%c#8%
[x1$1*-2](t1_2);
[x2$1*-2](t2_2);
[x3$1*-2](t3_2);
[x4$1*-2](t4_2);
[x5$1*-2](t5_2);
[x6$1*-2](t6_2);
[x7$1*-2](t7_2);
[x8$1*-2](t8_2);
[x9$1*-2](t9_2);
[x10$1*-2](t10_2);
[x11$1*-2](t11_2);
[x12$1*-2](t12_2);
[x13$1*-2](t13_2);
[x14$1*-2](t14_2);
```

Appendix B. EM Estimation with the compensatory QAMRM (N = 1000)

```
title:
montecarlo:
        NAMES = x1-x14;
        generate = x1-x14(1);
        categorical = x1-x14;
        genclasses = c(8);
        classes = c(8);
        nobs = 1000;
        seed = 3454367;
        nrep = 1000;

ANALYSIS:
        TYPE = MIXTURE;
       ALGORITHM=INTEGRATION;

model population:
        %overall%
       f BY x1-x14*1(1);
       [f@0];
       f@1;
        [c#1*0];
        [c#2*0];
        [c#3*0];
        [c#4*0];
        [c#5*0];
        [c#6*0];
        [c#7*0];

%c#1%
[x1$1*2](t1_1);
[x2$1*2](t2_1);
[x3$1*2](t3_1);
[x4$1*2](t4_1);
[x5$1*2](t5_1);
[x6$1*2](t6_1);
[x7$1*2](t7_1);
[x8$1*2](t8_1);
[x9$1*2](t9_1);
[x10$1*2](t10_1);
[x11$1*2](t11_1);
[x12$1*2](t12_1);
[x13$1*2](t13_1);
[x14$1*2](t14_1);
%c#2%
[x1$1*2](t1_1);
[x2$1*2](t2_1);
[x3$1*-2](t3_2);
[x4$1*2](t4_1);
[x5$1*1](t5_2);
[x6$1*1](t6_2);
[x7$1*1](t7_2);
[x8$1*2](t8_1);
[x9$1*2](t9_1);
[x10$1*-2](t10_2);
[x11$1*2](t11_1);
[x12$1*1](t12_2);
```

[x13$1*1](t13_2);
[x14$1*1](t14_2);
%c#3%
[x1$1*2](t1_1);
[x2$1*-2](t2_2);
[x3$1*2](t3_1);
[x4$1*1](t4_2);
[x5$1*2](t5_1);
[x6$1*1](t6_2);
[x7$1*1](t7_2);
[x8$1*2](t8_1);
[x9$1*-2](t9_2);
[x10$1*2](t10_1);
[x11$1*1](t11_2);
[x12$1*2](t12_1);
[x13$1*1](t13_2);
[x14$1*1](t14_2);
%c#4%
[x1$1*2](t1_1);
[x2$1*-2](t2_2);
[x3$1*-2](t3_2);
[x4$1*1](t4_2);
[x5$1*1](t5_2);
[x6$1*-2](t6_3);
[x7$1*-1](t7_3);
[x8$1*2](t8_1);
[x9$1*-2](t9_2);
[x10$1*-2](t10_2);
[x11$1*1](t11_2);
[x12$1*1](t12_2);
[x13$1*-2](t13_3);
[x14$1*-1](t14_3);
%c#5%
[x1$1*-2](t1_2);
[x2$1*2](t2_1);
[x3$1*2](t3_1);
[x4$1*1](t4_2);
[x5$1*1](t5_2);
[x6$1*2](t6_1);
[x7$1*1](t7_2);
[x8$1*-2](t8_2);
[x9$1*2](t9_1);
[x10$1*2](t10_1);
[x11$1*1](t11_2);
[x12$1*1](t12_2);
[x13$1*2](t13_1);
[x14$1*1](t14_2);
%c#6%
[x1$1*-2](t1_2);
[x2$1*2](t2_1);
[x3$1*-2](t3_2);
[x4$1*1](t4_2);
[x5$1*-2](t5_3);
[x6$1*1](t6_2);
[x7$1*-1](t7_3);
[x8$1*-2](t8_2);
[x9$1*2](t9_1);
[x10$1*-2](t10_2);

```
[x11$1*1](t11_2);
[x12$1*-2](t12_3);
[x13$1*1](t13_2);
[x14$1*-1](t14_3);
%c#7%
[x1$1*-2](t1_2);
[x2$1*-2](t2_2);
[x3$1*2](t3_1);
[x4$1*-2](t4_3);
[x5$1*1](t5_2);
[x6$1*1](t6_2);
[x7$1*-1](t7_3);
[x8$1*-2](t8_2);
[x9$1*-2](t9_2);
[x10$1*2](t10_1);
[x11$1*-2](t11_3);
[x12$1*1](t12_2);
[x13$1*1](t13_2);
[x14$1*-1](t14_3);
%c#8%
[x1$1*-2](t1_2);
[x2$1*-2](t2_2);
[x3$1*-2](t3_2);
[x4$1*-2](t4_3);
[x5$1*-2](t5_3);
[x6$1*-2](t6_3);
[x7$1*-2](t7_4);
[x8$1*-2](t8_2);
[x9$1*-2](t9_2);
[x10$1*-2](t10_2);
[x11$1*-2](t11_3);
[x12$1*-2](t12_3);
[x13$1*-2](t13_3);
[x14$1*-2](t14_4);

model:
     %overall%
    f BY x1-x14*1(1);
    [f@0];
    f@1;
     [c#1*0];
     [c#2*0];
     [c#3*0];
     [c#4*0];
     [c#5*0];
     [c#6*0];
     [c#7*0];

%c#1%
[x1$1*2](t1_1);
[x2$1*2](t2_1);
[x3$1*2](t3_1);
[x4$1*2](t4_1);
[x5$1*2](t5_1);
[x6$1*2](t6_1);
[x7$1*2](t7_1);
[x8$1*2](t8_1);
[x9$1*2](t9_1);
```

```
[x10$1*2](t10_1);
[x11$1*2](t11_1);
[x12$1*2](t12_1);
[x13$1*2](t13_1);
[x14$1*2](t14_1);
%c#2%
[x1$1*2](t1_1);
[x2$1*2](t2_1);
[x3$1*-2](t3_2);
[x4$1*2](t4_1);
[x5$1*1](t5_2);
[x6$1*1](t6_2);
[x7$1*1](t7_2);
[x8$1*2](t8_1);
[x9$1*2](t9_1);
[x10$1*-2](t10_2);
[x11$1*2](t11_1);
[x12$1*1](t12_2);
[x13$1*1](t13_2);
[x14$1*1](t14_2);
%c#3%
[x1$1*2](t1_1);
[x2$1*-2](t2_2);
[x3$1*2](t3_1);
[x4$1*1](t4_2);
[x5$1*2](t5_1);
[x6$1*1](t6_2);
[x7$1*1](t7_2);
[x8$1*2](t8_1);
[x9$1*-2](t9_2);
[x10$1*2](t10_1);
[x11$1*1](t11_2);
[x12$1*2](t12_1);
[x13$1*1](t13_2);
[x14$1*1](t14_2);
%c#4%
[x1$1*2](t1_1);
[x2$1*-2](t2_2);
[x3$1*-2](t3_2);
[x4$1*1](t4_2);
[x5$1*1](t5_2);
[x6$1*-2](t6_3);
[x7$1*-1](t7_3);
[x8$1*2](t8_1);
[x9$1*-2](t9_2);
[x10$1*-2](t10_2);
[x11$1*1](t11_2);
[x12$1*1](t12_2);
[x13$1*-2](t13_3);
[x14$1*-1](t14_3);
%c#5%
[x1$1*-2](t1_2);
[x2$1*2](t2_1);
[x3$1*2](t3_1);
[x4$1*1](t4_2);
[x5$1*1](t5_2);
[x6$1*2](t6_1);
[x7$1*1](t7_2);
```

```
[x8$1*-2](t8_2);
[x9$1*2](t9_1);
[x10$1*2](t10_1);
[x11$1*1](t11_2);
[x12$1*1](t12_2);
[x13$1*2](t13_1);
[x14$1*1](t14_2);
%c#6%
[x1$1*-2](t1_2);
[x2$1*2](t2_1);
[x3$1*-2](t3_2);
[x4$1*1](t4_2);
[x5$1*-2](t5_3);
[x6$1*1](t6_2);
[x7$1*-1](t7_3);
[x8$1*-2](t8_2);
[x9$1*2](t9_1);
[x10$1*-2](t10_2);
[x11$1*1](t11_2);
[x12$1*-2](t12_3);
[x13$1*1](t13_2);
[x14$1*-1](t14_3);
%c#7%
[x1$1*-2](t1_2);
[x2$1*-2](t2_2);
[x3$1*2](t3_1);
[x4$1*-2](t4_3);
[x5$1*1](t5_2);
[x6$1*1](t6_2);
[x7$1*-1](t7_3);
[x8$1*-2](t8_2);
[x9$1*-2](t9_2);
[x10$1*2](t10_1);
[x11$1*-2](t11_3);
[x12$1*1](t12_2);
[x13$1*1](t13_2);
[x14$1*-1](t14_3);
%c#8%
[x1$1*-2](t1_2);
[x2$1*-2](t2_2);
[x3$1*-2](t3_2);
[x4$1*-2](t4_3);
[x5$1*-2](t5_3);
[x6$1*-2](t6_3);
[x7$1*-2](t7_4);
[x8$1*-2](t8_2);
[x9$1*-2](t9_2);
[x10$1*-2](t10_2);
[x11$1*-2](t11_3);
[x12$1*-2](t12_3);
[x13$1*-2](t13_3);
[x14$1*-2](t14_4);
```

Appendix C. Hierarchical QAMRM Analysis with the ECPE Data

```
TITLE: Hierarchical QAMRM
DATA: FILE IS ecpedata.dat;
VARIABLE:
NAMES = X1-X28 ID P1-P8 N;
        USEVARIABLE = X1-X28;
        CATEGORICAL = X1-X28;
        CLASSES = C(4);
ANALYSIS:
    TYPE=MIXTURE;
    ALGORITHM=INTEGRATION;
    STARTS=0;
    INTEGRATION = 15;
    PROCESSORS=4;
OUTPUT:
    TECH1 TECH5 TECH8 TECH10;
MODEL:
    %OVERALL%
     F BY X1-X28*(1);
     [f@0];
     f@1;
    [C#1] (M1); !profile [000]
    [C#2] (M2); !profile [001]
    [C#3] (M3); !profile [011]

    %c#1% !column #1 of item respond function labels table for profile [000]
    [X1$1] (T1_1); !item 1 _ threshold 1
    [X2$1] (T2_1); !item 1 _ threshold 1
    [X3$1] (T3_1); !item 1 _ threshold 1
    [X4$1] (T4_1); !item 1 _ threshold 1
    [X5$1] (T5_1); !item 1 _ threshold 1
    [X6$1] (T6_1); !item 2 _ threshold 1
    [X7$1] (T7_1); !item 2 _ threshold 1
    [X8$1] (T8_1); !item 2 _ threshold 1
    [X9$1] (T9_1); !item 2 _ threshold 1
    [X10$1] (T10_1); !item 2 _ threshold 1
    [X11$1] (T11_1); !item 3 _ threshold 1
    [X12$1] (T12_1); !item 3 _ threshold 1
    [X13$1] (T13_1); !item 3 _ threshold 1
    [X14$1] (T14_1); !item 3 _ threshold 1
    [X15$1] (T15_1); !item 3 _ threshold 1
    [X16$1] (T16_1); !item 4 _ threshold 1
    [X17$1] (T17_1); !item 4 _ threshold 1
    [X18$1] (T18_1); !item 4 _ threshold 1
    [X19$1] (T19_1); !item 4 _ threshold 1
    [X20$1] (T20_1); !item 4 _ threshold 1
    [X21$1] (T21_1); !item 5 _ threshold 1
    [X22$1] (T22_1); !item 5 _ threshold 1
    [X23$1] (T23_1); !item 5 _ threshold 1
    [X24$1] (T24_1); !item 5 _ threshold 1
    [X25$1] (T25_1); !item 5 _ threshold 1
    [X26$1] (T26_1); !item 6 _ threshold 1
    [X27$1] (T27_1); !item 6 _ threshold 1
    [X28$1] (T28_1); !item 6 _ threshold 1
    %c#2% !column #2 of item respond function labels table for profile [001]
    [X1$1] (T1_1); !item 1 _ threshold 2
    [X2$1] (T2_1); !item 1 _ threshold 2
    [X3$1] (T3_2); !item 1 _ threshold 2
```

[X4$1] (T4_2); !item 1 _ threshold 2
[X5$1] (T5_2); !item 1 _ threshold 2
[X6$1] (T6_2); !item 2 _ threshold 1
[X7$1] (T7_2); !item 2 _ threshold 1
[X8$1] (T8_1); !item 2 _ threshold 1
[X9$1] (T9_2); !item 2 _ threshold 1
[X10$1] (T10_1); !item 2 _ threshold 1
[X11$1] (T11_2); !item 3 _ threshold 1
[X12$1] (T12_2); !item 3 _ threshold 1
[X13$1] (T13_1); !item 3 _ threshold 1
[X14$1] (T14_1); !item 3 _ threshold 1
[X15$1] (T15_2); !item 3 _ threshold 1
[X16$1] (T16_2); !item 4 _ threshold 3
[X17$1] (T17_2); !item 4 _ threshold 3
[X18$1] (T18_2); !item 4 _ threshold 3
[X19$1] (T19_2); !item 4 _ threshold 3
[X20$1] (T20_2); !item 4 _ threshold 3
[X21$1] (T21_2); !item 5 _ threshold 3
[X22$1] (T22_2); !item 5 _ threshold 3
[X23$1] (T23_1); !item 5 _ threshold 3
[X24$1] (T24_1); !item 5 _ threshold 3
[X25$1] (T25_1); !item 5 _ threshold 3
[X26$1] (T26_2); !item 6 _ threshold 1
[X27$1] (T27_1); !item 6 _ threshold 1
[X28$1] (T28_2); !item 6 _ threshold 1

%c#3% !column #3 of item respond function labels table for profile [011]
[X1$1] (T1_2); !item 1 _ threshold 2
[X2$1] (T2_2); !item 1 _ threshold 2
[X3$1] (T3_2); !item 1 _ threshold 2
[X4$1] (T4_2); !item 1 _ threshold 2
[X5$1] (T5_2); !item 1 _ threshold 2
[X6$1] (T6_2); !item 2 _ threshold 2
[X7$1] (T7_2); !item 2 _ threshold 2
[X8$1] (T8_2); !item 2 _ threshold 2
[X9$1] (T9_2); !item 2 _ threshold 2
[X10$1] (T10_1); !item 2 _ threshold 2
[X11$1] (T11_2); !item 3 _ threshold 1
[X12$1] (T12_2); !item 3 _ threshold 1
[X13$1] (T13_1); !item 3 _ threshold 1
[X14$1] (T14_1); !item 3 _ threshold 1
[X15$1] (T15_2); !item 3 _ threshold 1
[X16$1] (T16_2); !item 4 _ threshold 4
[X17$1] (T17_3); !item 4 _ threshold 4
[X18$1] (T18_2); !item 4 _ threshold 4
[X19$1] (T19_2); !item 4 _ threshold 4
[X20$1] (T20_2); !item 4 _ threshold 4
[X21$1] (T21_2); !item 5 _ threshold 3
[X22$1] (T22_2); !item 5 _ threshold 3
[X23$1] (T23_2); !item 5 _ threshold 3
[X24$1] (T24_2); !item 5 _ threshold 3
[X25$1] (T25_1); !item 5 _ threshold 3
[X26$1] (T26_2); !item 6 _ threshold 3
[X27$1] (T27_1); !item 6 _ threshold 3
[X28$1] (T28_2); !item 6 _ threshold 3

%c#4% !column #4 of item respond function labels table for profile [111]
[X1$1] (T1_3); !item 1 _ threshold 2

```
        [X2$1] (T2_2); !item 1 _ threshold 2
        [X3$1] (T3_3); !item 1 _ threshold 2
        [X4$1] (T4_2); !item 1 _ threshold 2
        [X5$1] (T5_2); !item 1 _ threshold 2
        [X6$1] (T6_2); !item 2 _ threshold 2
        [X7$1] (T7_3); !item 2 _ threshold 2
        [X8$1] (T8_2); !item 2 _ threshold 2
        [X9$1] (T9_2); !item 2 _ threshold 2
        [X10$1] (T10_2); !item 2 _ threshold 2
        [X11$1] (T11_3); !item 3 _ threshold 2
        [X12$1] (T12_3); !item 3 _ threshold 2
        [X13$1] (T13_2); !item 3 _ threshold 2
        [X14$1] (T14_2); !item 3 _ threshold 2
        [X15$1] (T15_2); !item 3 _ threshold 2
        [X16$1] (T16_3); !item 4 _ threshold 4
        [X17$1] (T17_3); !item 4 _ threshold 4
        [X18$1] (T18_2); !item 4 _ threshold 4
        [X19$1] (T19_2); !item 4 _ threshold 4
        [X20$1] (T20_3); !item 4 _ threshold 4
        [X21$1] (T21_3); !item 5 _ threshold 4
        [X22$1] (T22_2); !item 5 _ threshold 4
        [X23$1] (T23_2); !item 5 _ threshold 4
        [X24$1] (T24_2); !item 5 _ threshold 4
        [X25$1] (T25_2); !item 5 _ threshold 4
        [X26$1] (T26_2); !item 6 _ threshold 4
        [X27$1] (T27_2); !item 6 _ threshold 4
        [X28$1] (T28_2); !item 6 _ threshold 4

MODEL CONSTRAINT: ! follow Templin and Bradshaw(2014) to set parameter constraint by
                                        Q-matrix
NEW(L1_0*1.5 L1_12*-2 L1_221*-1);
        T1_1=(L1_0);
        T1_2=(L1_0+L1_12);
        T1_3=(L1_0+L1_12+L1_221);
        L1_12<0; L1_221<0;
NEW(L2_0*1 L2_12*-2);
        T2_1=(L2_0);
        T2_2=(L2_0+L2_12);
        L2_12<0;
NEW(L3_0*1.5 L3_13*-2 L3_231*-1);
        T3_1=(L3_0);
        T3_2=(L3_0+L3_13);
        T3_3=(L3_0+L3_13+L3_231);
        L3_13<0; L3_231<0;
NEW(L4_0*1 L4_13*-2);
        T4_1=(L4_0);
        T4_2=(L4_0+L4_13);
        L4_13<0;
NEW(L5_0*1 L5_13*-2);
        T5_1=(L5_0);
        T5_2=(L5_0+L5_13);
        L5_13<0;
NEW(L6_0*1 L6_13*-2);
        T6_1=(L6_0);
        T6_2=(L6_0+L6_13);
        L6_13<0;
NEW(L7_0*1.5 L7_13*-2 L7_231*-1);
        T7_1=(L7_0);
```

```
        T7_2=(L7_0+L7_13);
        T7_3=(L7_0+L7_13+L7_231);
        L7_13<0; L7_231<0;
NEW(L8_0*1 L8_12*-2);
        T8_1=(L8_0);
        T8_2=(L8_0+L8_12);
        L8_12<0;
NEW(L9_0*1 L9_13*-2);
        T9_1=(L9_0);
        T9_2=(L9_0+L9_13);
        L9_13<0;
NEW(L10_0*1 L10_11*-2);
        T10_1=(L10_0);
        T10_2=(L10_0+L10_11);
        L10_11<0;
NEW(L11_0*1.5 L11_13*-2 L11_231*-1);
        T11_1=(L11_0);
        T11_2=(L11_0+L11_13);
        T11_3=(L11_0+L11_13+L11_231);
        L11_13<0; L11_231<0;
NEW(L12_0*1.5 L12_13*-2 L12_231*-1);
        T12_1=(L12_0);
        T12_2=(L12_0+L12_13);
        T12_3=(L12_0+L12_13+L12_231);
        L12_13<0; L12_231<0;
NEW(L13_0*1 L13_11*-2);
        T13_1=(L13_0);
        T13_2=(L13_0+L13_11);
        L13_11<0;
NEW(L14_0*1 L14_11*-2);
        T14_1=(L14_0);
        T14_2=(L14_0+L14_11);
        L14_11<0;
NEW(L15_0*1 L15_13*-2);
        T15_1=(L15_0);
        T15_2=(L15_0+L15_13);
        L15_13<0;
NEW(L16_0*1.5 L16_13*-2 L16_231*-1);
        T16_1=(L16_0);
        T16_2=(L16_0+L16_13);
        T16_3=(L16_0+L16_13+L16_231);
        L16_13<0; L16_231<0;
NEW(L17_0*1.5 L17_13*-2 L17_232*-1);
        T17_1=(L17_0);
        T17_2=(L17_0+L17_13);
        T17_3=(L17_0+L17_13+L17_232);
        L17_13<0; L17_232<0;
NEW(L18_0*1 L18_13*-2);
        T18_1=(L18_0);
        T18_2=(L18_0+L18_13);
        L18_13<0;
NEW(L19_0*1 L19_13*-2);
        T19_1=(L19_0);
        T19_2=(L19_0+L19_13);
        L19_13<0;
NEW(L20_0*1.5 L20_13*-2 L20_231*-1);
        T20_1=(L20_0);
        T20_2=(L20_0+L20_13);
```

```
        T20_3=(L20_0+L20_13+L20_231);
        L20_13<0; L20_231<0;
NEW(L21_0*1.5 L21_13*-2 L21_231*-1);
        T21_1=(L21_0);
        T21_2=(L21_0+L21_13);
        T21_3=(L21_0+L21_13+L21_231);
        L21_13<0; L21_231<0;
NEW(L22_0*1 L22_13*-2);
        T22_1=(L22_0);
        T22_2=(L22_0+L22_13);
        L22_13<0;
NEW(L23_0*1 L23_12*-2);
        T23_1=(L23_0);
        T23_2=(L23_0+L23_12);
        L23_12<0;
NEW(L24_0*1 L24_12*-2);
        T24_1=(L24_0);
        T24_2=(L24_0+L24_12);
        L24_12<0;
NEW(L25_0*1 L25_11*-2);
        T25_1=(L25_0);
        T25_2=(L25_0+L25_11);
        L25_11<0;
NEW(L26_0*1 L26_13*-2);
        T26_1=(L26_0);
        T26_2=(L26_0+L26_13);
        L26_13<0;
NEW(L27_0*1 L27_11*-2);
        T27_1=(L27_0);
        T27_2=(L27_0+L27_11);
        L27_11<0;
NEW(L28_0*1 L28_13*-2);
        T28_1=(L28_0);
        T28_2=(L28_0+L28_13);
        L28_13<0;
```
